# Validation of the RRE-90 Scale to Predict Stroke Risk after Transient Symptoms with Infarction: A Prospective Cohort Study

**DOI:** 10.1371/journal.pone.0137425

**Published:** 2015-09-22

**Authors:** Bo Song, Lulu Pei, Hui Fang, Lu Zhao, Yuan Gao, Yuanyuan Wang, Song Tan, Yuming Xu

**Affiliations:** 1 Department of Neurology, the First Affiliated Hospital of Zhengzhou University, Zhengzhou, Henan Province, China; 2 Department of Neurology, the Sichuan Provincial People's Hospital, Chengdu, Sichuan Province, China; National Cardiovascular Center Hospital, JAPAN

## Abstract

**Background and Purpose:**

The risk of stroke after a transient ischemic attack (TIA) for patients with a positive diffusion-weighted image (DWI), i.e., transient symptoms with infarction (TSI), is much higher than for those with a negative DWI. The aim of this study was to validate the predictive value of a web-based recurrence risk estimator (RRE; http://www.nmr.mgh.harvard.edu/RRE/) of TSI.

**Methods:**

Data from the prospective hospital-based TIA database of the First Affiliated Hospital of Zhengzhou University were analyzed. The RRE and ABCD^2^ scores were calculated within 7 days of symptom onset. The predictive outcome was ischemic stroke occurrence at 90 days. The receiver-operating characteristics curves were plotted, and the predictive value of the two models was assessed by computing the C statistics.

**Results:**

A total of 221 eligible patients were prospectively enrolled, of whom 46 (20.81%) experienced a stroke within 90 days. The 90-day stroke risk in high-risk TSI patients (RRE ≥4) was 3.406-fold greater than in those at low risk (P <0.001). The C statistic of RRE (0.681; 95% confidence interval [CI], 0.592–0.771) was statistically higher than that of ABCD^2^ score (0.546; 95% CI, 0.454–0.638; Z = 2.115; P = 0.0344) at 90 days.

**Conclusion:**

The RRE score had a higher predictive value than the ABCD^2^ score for assessing the 90-day risk of stroke after TSI.

## Introduction

Transient ischemic attack (TIA) is a common manifestation of acute cerebrovascular disease [1–3]. Patients with TIA are at high risk of early stroke, especially those with a high signal on diffusion-weighted imaging (DWI) maps [3–8]. According to previous studies, DWI abnormalities are present in approximately one-third of TIA patients [4,9–11]. TIA with an abnormal DWI result is defined as transient symptoms with infarction (TSI) [12]. Nevertheless, because the majority of patients will not have a subsequent stroke after TSI, a tool designed to identify patients at high risk of early stroke, in whom urgent evaluation and intervention is most justified, would be of tremendous benefit. No prognostic score has been specifically developed for TSI. A retrospective study showed that the RRE-90 (90-day Recurrence Risk Estimator) scale [13], previously designed for the prediction of the recurrence of stroke, had a high predictive value in predicting 7-day stroke risk after TSI [14]. If further validated for 90-day risk prediction, RRE could be applied to any patient with infarction on DWI maps, regardless of transient or persistent symptoms. Hence, it is crucial to further prospectively validate the RRE score.

The aim of this hospital-based study is to prospectively validate the 90-day predictive value of the RRE-90 score in TSI patients, and to triage TSI patients more precisely to promote active treatment with secondary prevention strategies.

## Materials and Methods

### Patient Selection

The TIA database of the First Affiliated Hospital of Zhengzhou University prospectively enrolled consecutive hospitalized patients with a diagnosis of TIA (elapsed time from last episode to registry <7 days) from 2010, as stated previously [15,16]. TIA was diagnosed based on World Health Organization (WHO) diagnostic criteria [17], which define a TIA as an acute loss of focal cerebral or ocular dysfunction lasting less than 24 h attributed to embolic or thrombotic vascular diseases. The TSI data were taken from the database in which TIA showed an abnormally high signal on DWI.

Those ruled out by exclusion criteria included patients with unclear clinical information; patients who refused to participate in the research and who did not complete the follow-up protocol; patients with severe disorders such as cancer and hepatic disease; patients who received endovascular therapy and underwent surgery after Moyamoya disease or patent foramen ovale; and patients with unavailable RRE-90 scores.

Trained physicians recorded all detailed baseline data of enrolled TIA patients by using paper case report forms, including demographics, clinical features, imaging features, ABCD^2^ score, RRE-90 score, etiologic stroke subtype using the automated Causative Classification of Stroke System (CCS) [18], and treatment for secondary prevention. DWI was performed routinely in all TIA patients without contraindications to magnetic resonance imaging (MRI) within 7 days of symptom onset. All of the images were assessed by two professional neurologists.

### Ethics Statement

This study was approved by the Ethics Committee of the First Affiliated Hospital of Zhengzhou University. All patients or their legally authorized representatives signed an informed consent form.

### RRE-90 Scale and ABCD^2^ Score

The 6-point Web-based RRE score (http://www.nmr.mgh.harvard.edu/RRE/) (Table 1) was originally developed to predict the 14- and 90-day risk of recurrence in patients with ischemic stroke [13]. The ABCD^2^ score [19] is a 7-point scale consisting of five clinical variables: age, blood pressure, clinical features, duration of symptoms, and history of diabetes mellitus.

**Table 1:** RRE-90 scale.

	RRE
**History of TIA or stroke within the preceding month of index stroke**	1
**Multiple acute infarction**	1
**Simultaneous infarcts in different circulations**	1
**Multiple infarcts of different ages**	1
**Isolated cortical infarcts**	1
**CCS etiologic stroke subtype**	
**Large Artery Atherosclerosis**	1
**Cardioaortic Embolism**	0
**Small Artery Occlusion**	0
**Other Causes**	1
**Undetermined Causes**	0

The components of the RRE score showed the rules the study achieved the raw data.

TIA indicates transient ischemic attack.

### Definitions

TIA with DWI evidence of acute infarction was defined as TSI [12]. The index TSI was defined as the most recent assessment by a stroke specialist. Duration of symptoms was calculated from the last time the patient was known to be well. Abnormally acute DWI hyperintensity was defined as a lesion consistent with acute cerebral ischemia. Carotid and intracranial artery stenosis was defined as ≥50% narrowing in the lumen of the intracranial or carotid artery responsible for the neurological symptoms. The evaluation of vessels was based on: carotid artery ultrasonography; transcranial Doppler; magnetic resonance, computed tomographic or digital subtraction angiography; and the percentage of stenosis, calculated using the North American Symptomatic Carotid Endarterectomy Trial (NASCET) method [20]. Stroke etiology was classified by the CCS [18], a Web-based system consisting of a questionnaire-style classification scheme for ischemic stroke (http://ccs.martinos.org). Information on demography and vascular risk factors including history of hypertension, hyperlipidemia, diabetes mellitus, coronary heart disease, and atrial fibrillation was obtained from patients’ self-reports with past medical records or treatment data. “Current smoker” indicated smoking within 6 months before registration.

### Predictive Outcome

All registered patients were followed up via telephone interview by a neurologist blinded to the clinical information and prognostic scores. The end point was occurrence of ischemic stroke. All patients who were suspected of having a stroke were followed up via face-to-face interview. Stroke was defined as the sudden onset of neurological symptoms that persisted for ≥24 h based on the WHO criteria [17].

### Statistical Analysis

The Chi-square test (Mann–Whitney U test) was used to determine the correlation between baseline categorical variables, and a t-test was used for continuous variables. The cumulative incidence was calculated by Kaplan–Meier analysis. The Cochran–Armitage trend test was performed for each score of the RRE, and the risk of stroke (stratified according to the score) was compared by the log-rank test. Cox proportional hazards regression modeling was conducted to determine the risk factors associated with recurrent stroke. Associations were presented as hazard ratio (HR) with corresponding 95% confidence interval (CI). Multivariate analysis was performed using stroke as the dependent variable, with inclusion of independent variables associated at the P<0.1 level in univariate analysis (Forward: LR). P<0.05 was considered statistically significant in the final multivariate analyses. The receiver-operating characteristics (ROC) curves were plotted, and C statistics calculated as a measure of predictive ability. The optimal cut-off point was chosen as the point with maximum Youden index. The Z test was used to compare C statistics. All statistical analyses were performed using SPSS 16.0.

## Results

**Fig 1:**
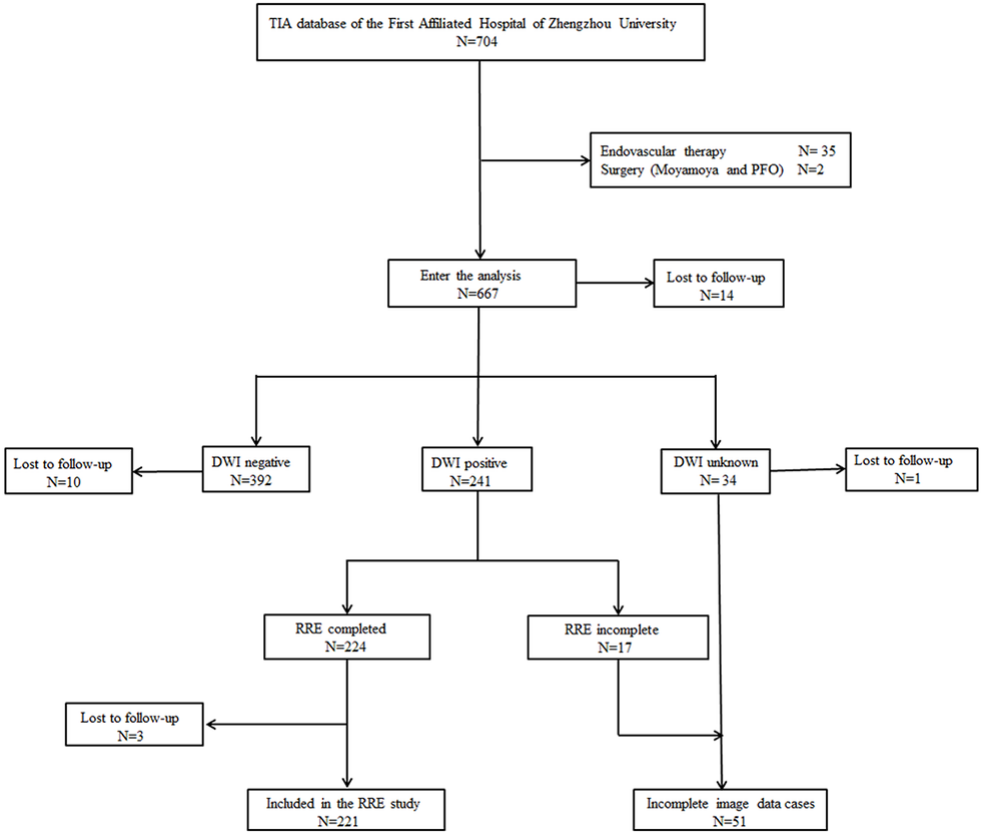
Flow diagram defining the potentially eligible TSI patients. The flow diagram was used to illustrate how the study population was selected.

A series of 704 consecutive patients hospitalized in the First Affiliated Hospital of Zhengzhou University between 2010 and 2014 with a diagnosis of definite TIA were prospectively enrolled. Excluded were 35 patients who had received endovascular therapy and two who had undergone surgery because of Moyamoya disease and patent foramen ovale. Of the remaining 667 patients, 392 showed negative results on DWI, 241 showed positive results (TSI), and 34 had no MRI data. The RRE study defined the Image-incomplete group (n = 51, 7.6%) as patients with missing MRI data (n = 34) or TSI patients who did not achieve an RRE-90 score (n = 17, 7.1%). Comparison of baseline characteristics of the Image-complete and Image-incomplete groups showed no significant differences (Table 2). Fig 1 shows the flow diagram defining the potentially eligible TSI patients. The 90-day stroke risk of DWI-positive and DWI-negative patients is presented in Table 3.

**Table 2:** Clinical characteristics of image-complete group and image-incomplete group.

	Image-complete	Image-incomplete	P Value
	N (%)	N (%)	
**Female**	258 (41.9)	20 (39.2)	0.711
**Age≥60 y**	282 (45.8)	20 (39.2)	0.366
**History of stroke**	114 (18.5)	6 (11.8)	0.229
**Coronary artery disease**	65 (10.6)	8 (15.7)	0.259
**Atrial fibrillation**	11 (1.8)	0 (0)	—
**Hypertension**	339 (55.0)	26 (51.0)	0.577
**Hyperlipidemia**	86 (14.0)	5 (9.8)	0.406
**Diabetes mellitus**	88 (14.3)	9 (17.6)	0.513
**Antiplatelet history**	132 (21.4)	10 (19.6)	0.760
**Anticoagulation history**	4 (0.6)	0 (0)	—
**Lipid-lowering history**	84 (13.6)	9 (17.6)	0.427
**Antidiabetics history**	73 (11.9)	8 (15.7)	0.421
**Antihypertensive history**	242 (39.3)	20 (39.2)	0.992
**ABCD** ^**2**^ **score≥4**	260 (42.2)	22 (43.1)	0.897
**Current smoker**	153 (24.8)	14 (27.5)	0.679

The comparison of baseline characteristics of Image-complete group and Image-incomplete group showed no significant differences (P>0.05).

**Table 3:** The risk of stroke for DWI positive patients (TSI) and DWI negative patients.

DWI	Patient No.	Follow-up No.	Stroke No.	Risk, % (95% CI)
**Positive**	241	238	50	21.01(15.83–26.19)
**Negative**	392	382	19	4.97(2.79–7.15)
**Total**	633	620	69	11.13(8.65–13.61)

Stroke risk for TSI patients was substantially higher than DWI negative patients (P<0.01).

CI indicates confidence interval.

The proportion of TSI accounted for 38.1%. A total of 224 eligible patients were enrolled after excluding 17 subjects with no RRE score. Three subjects (1.3%) were lost to follow-up at 3 months in the RRE study. The average age of the TSI patients was 57.48 ± 12.72 years (range, 17–87), and 87 (39.4%) were female. In the RRE study, 18 (8.14%), 26 (11.76%), 40 (18.10%) and 46 (20.81%) patients experienced a stroke within 2 days, 7 days, 30 days and 90 days, respectively.

**Fig 2:**
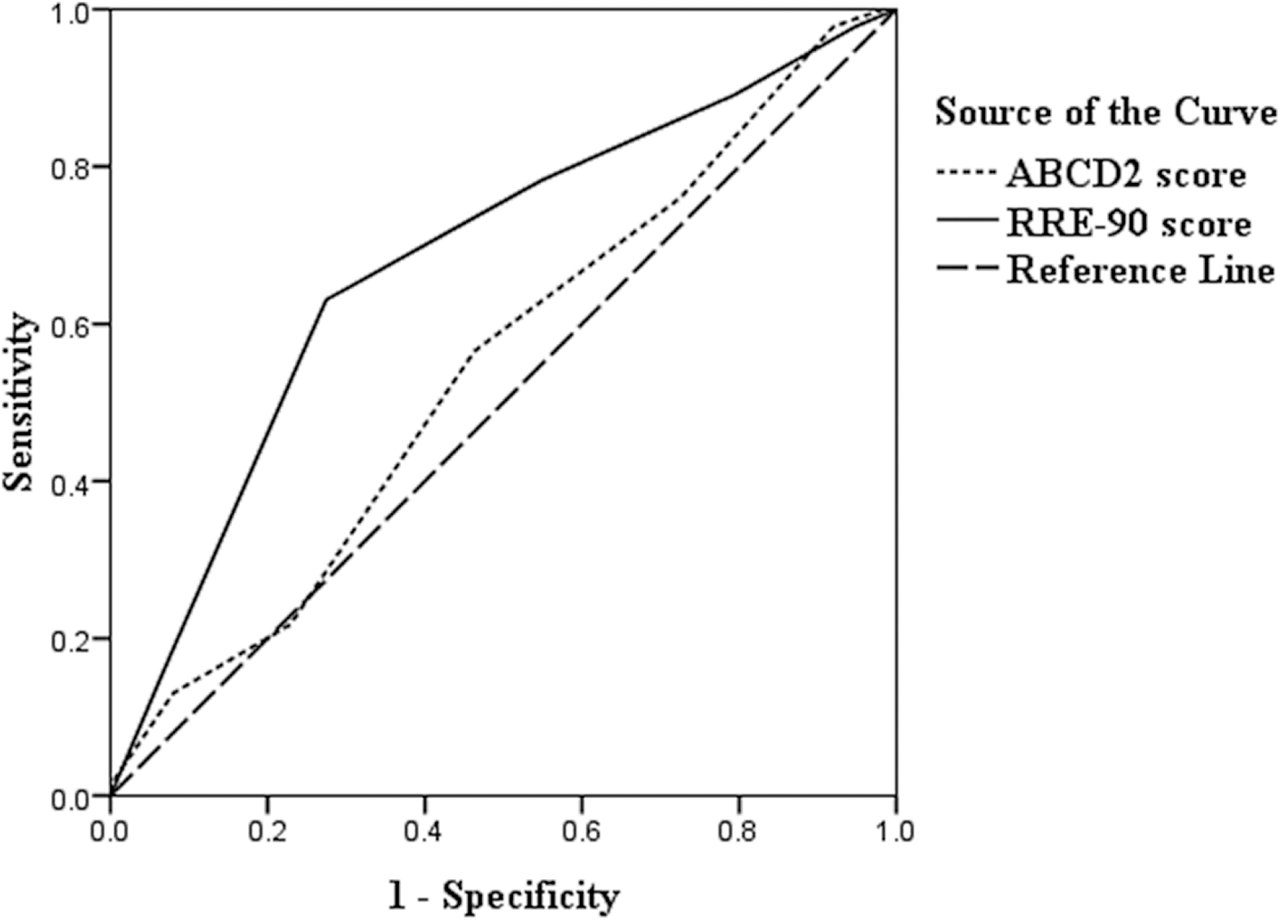
Ninety-day receiver-operating characteristic curves as a predictive value of the RRE score and ABCD^2^ score. The RRE score showed better discrimination (Z = 2.115, P = 0.0344) with an area under the receiver operating characteristic curve of 0.681 (95%CI, 0.592–0.771) than ABCD^2^ score (C statistics = 0.546; 95% CI, 0.454–0.638).

Fig 2 shows the ROC curve for predicting the 90-day risk of stroke in patients with TSI. The C statistic was 0.681 (95% CI, 0.592–0.771) by the RRE score and 0.546 (95% CI, 0.454–0.638) by the ABCD^2^ score. Z-testing revealed that the C statistic for the RRE score was higher than that for the ABCD^2^ score (Z = 2.115; P = 0.0344). The sensitivity, specificity, positive predictive value, negative predictive value and Youden index for each individual point were listed in Table 4. TSI patients were divided into low RRE group (0–3) and high RRE group (4–6) based on optimal cut-off point.

**Table 4:** Discrimination abilities of each point about the RRE score.

Score	Sensitivity	Specificity	PPV	NPV	Youden index
**0**	0.100	0.000	0.208	NA	0
**1**	0.978	0.051	0.213	0.900	0.029
**2**	0.891	0.206	0.228	0/878	0.097
**3**	0.783	0.452	0.273	0.888	0.235
**4**	0.630	0.726	0.377	0.882	0.356*
**5**	0.174	0.926	0.381	0.810	0.100
**6**	0.000	0.100	NA	0.792	0

TSI patients were divided into low RRE group (0–3) and high RRE group (4–6) based on optimal cut-off point which represents maximum Youden index.

PPV indicates positive predictive value; NPV indicates negativve predictive value; NA indicates not applicable.

*maximumYouden index.

**Fig 3:**
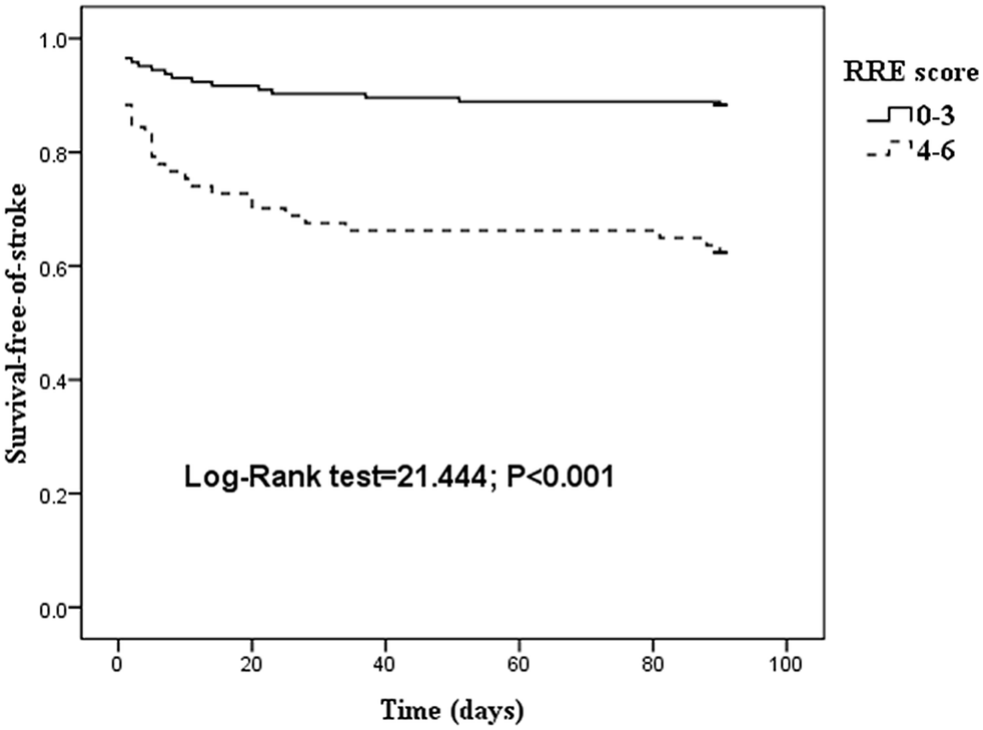
Kaplan–Meier curves of patients stratified according to the RRE score. Cum indicates cumulative. The Kaplan–Meier curves showed a significant difference between the low- (RRE<4) and high-risk (RRE≥4) categories (log-rank test = 21.444, P<0.001).

The survival–free-of-stroke curve categorized by the RRE score is shown in Fig 3. There was a significant difference between the low-risk (RRE <4) and high-risk (RRE ≥4) categories when overall comparisons were made (log-rank test = 21.444, P <0.001). A linear trend for occurrence rates was observed with the Cochran–Armitage trend test (Z = 3.6907, P = 0.0002; Table 5).

**Table 5:** The risk for stroke of each RRE score after TSI.

Score	Patient No	Stroke No.	Risk, % (95%)
**0**	10	1	10.00(0.25–55.72)
**1**	31	4	12.90(3.52–33.04)
**2**	48	5	10.42(3.38–24.31)
**3**	55	7	12.73(5.12–26.22)
**4**	56	21	37.50(23.21–57.32)
**5**	21	8	38.10(16.45–75.06)
**6**	0	0	0
**Total**	221	46	20.8 (15.24–27.76)

A linear trend for occurrence rates was observed with the Cochran–Armitage trend test (Z = 3.6907, P = 0.0002).

CI indicates confidence interval.

In the initial univariate Cox regression analyses (Table 6), the association of clinical features, RRE scores ≥4, ABCD^2^ score ≥4, stroke risk factors, and medications after discharge with the risk of subsequent stroke was investigated. The variables selected for entry into the final multivariate model (P<0.1) were: RRE ≥4, intracranial artery stenosis, use of antiplatelet medications after discharge and lipid-lowering medications after discharge. These variables were analyzed in the multivariate model and then removed by the Forward: LR regression procedure. The final multivariate analyses (Table 7) revealed that RRE ≥4 (HR 3.406; 95% CI, 1.854–6.257) and use of lipid-lowering agents after discharge (HR 7.131; 95% CI, 1.725–29.476) were independent predictors of stroke risk. More specifically, the 90-day stroke risk in high-risk TSI patients was 3.406-fold greater than in those at low risk (P <0.001), after adjusting for potential confounders.

**Table 6:** Univariate analysis of variables associated with stroke occurrence by 90 days.

		Stroke occurrence by 90 d		
	All patients	No (n = 175)	Yes (n = 46)	P Value
**Women**	87 (39.4)	64 (36.6)	23 (50.0)	0.115
**Age≥60y**	103 (46.6)	79 (45.1)	24 (52.2)	0.359
**History of stroke**	53 (24.0)	42 (24.0)	11 (23.9)	0.916
**Coronary heart disease**	20 (9.0)	18 (10.3)	2 (4.3)	0.219
**Atrial fibrillation**	6 (2.7)	5 (2.9)	1 (2.2)	0.843
**Hypertension**	134 (60.0)	103 (58.9)	31 (67.4)	0.303
**Hyperlipidemia**	30 (13.6)	24 (13.7)	6 (13.0)	0.877
**Diabetes mellitus**	33 (14.9)	26 (14.9)	7 (15.2)	0.980
**ABCD** ^**2**^ **≥ 4**	107 (48.4)	81 (46.3)	26 (56.5)	0.195
**RRE≥ 4**	77 (34.8)	48 (27.4)	29 (63.0)	<0.001
**Artery stenosis**				
**Carotid**	29 (13.1)	23 (13.4)	6 (13.3)	0.975
**Intracranial artery**	113 (51.1)	83 (47.7)	30 (68.2)	0.020
**Timing of DWI ≤72 h**	166 (75.1)	133 (76.0)	33 (71.7)	0.569
**Current smoker**	67 (30.3)	52 (29.7)	15(32.6)	0.643
**Secondary prevention**				
**Antiplatelet**	195 (88.2)	150 (85.7)	45 (97.8)	0.061
**Anticoagulant**	3 (1.4)	3 (1.7)	0 (0)	—
**Antihypertensive**	96 (43.4)	71 (40.6)	25 (54.3)	0.109
**Lipid-lowering**	168 (76.0)	125 (71.4)	43 (93.5)	0.006
**Antidiabetics**	29 (13.1)	22 (12.6)	7 (15.2)	0.615

Univariate analysis showed risk factors associated with stroke occurrence (P<0.1)

**Table 7:** Multivariate cox regression analysis for stroke occurrence by 90 days.

	Recurrence	No Recurrence	HR (95%CI)	P Value
**RRE≥ 4**	48 (27.4)	29 (63.0)	3.406(1.854–6.257)	<0.001
**Lipid-lowering**	43 (93.5)	125 (71.4)	7.131(1.725–29.476)	0.007

Multivariate cox regression analysis showed the independent risk factors (P<0.05).

HR indicates hazard ratio.

## Discussion

In our cohort, the proportion of DWI-positive subjects (38.1%) is consistent with that of 34.3% reported in a meta-analysis [11] comprising 47 papers and 9,078 patients (95% CI, 30.5–38.4%; range, 9–67%; I^2^ = 89.3%). In this study, the 90-day ischemic stroke rate was 21.01% (95% CI, 15.83–26.19%) in patients with TSI, which was substantially higher than the stroke incidence documented in previous studies [7,10,21]. Differences in ethnic factors and demographics, in addition to hospital-related factors, may explain the variations between studies. Correspondingly, the stroke risk for TIA without infarction was 4.97% (95% CI, 2.79–7.15%), which was substantially lower than TSI (P<0.01, Table 3). Several published data on patients with acute brain ischemia suggest that TIA without infarction and TSI differ in their short-term prognosis and that TSI is an independent entity, different from ischemic stroke and TIA [7,12,22]. Therefore, a new tissue-based definition was proposed by the American Heart Association (2009), which defines TIA as a transient episode of neurological dysfunction caused by focal brain, spinal cord, or retinal ischemia, without acute infarction [23]. Considering the higher stroke risk after TSI, our study supports the viewpoint that TSI may be an independent entity, different from pure TIA (TIA with a negative DWI result). The fact that TIA patients without infarction experienced non-ischemic events such as epilepsy, migraine, seizures, syncope, or somatoform disease might explain why patients with TIA but without infarction are at lower risk of stroke. Given the greater risk of imminent stroke for TSI, physicians should be alert that patients with such transient events require urgent care.

In this study, the C statistic for the ABCD^2^ score was 0.546 (95% CI, 0.454–0.638) at 90 days, no different from predictions based on chance alone. A multicenter cohort study indicated that tissue-positive events with low ABCD^2^ scores and tissue-negative events with high ABCD^2^ scores had similar stroke risks, especially after a 90-day follow-up [6]. A systematic review and meta-analysis [24] showed a non-significant trend towards overprediction of stroke in all risk categories at 90 days for ABCD^2^ scores. Meta-analysis [25] showed that the performance of the ABCD^2^ score was poor in identifying both high-risk TIA patients and those at modest to low risk. Some studies have questioned the prognostic value of the ABCD^2^ score because of its failure to take atrial fibrillation, etiology, and imaging findings into account. The ABCD^2^ score should especially be used with caution in young patients and those with other risk factors for recurrent stroke, including a positive DWI lesion [26,27].

This study revealed that the risk of recurrent stroke had a significant linear correlation with increasing RRE score (Table 5). In the final multivariate model, RRE ≥4 and use of lipid-lowering medication after discharge were statistically significant. As shown in Table 6, the patients with stroke were much older and had much more risk factors (hypertension, intracranial artery stenosis and smoking) than those without stroke, though the difference had no statistics significance. Although, active therapies have been given to those older patients, ischemic events could not be avoided absolutely. The reasons above could explain their greater use of lipid-lowering agents after discharge in comparison with the no-recurrence group. Moreover, our study showed that the RRE score had a higher predictive value than the ABCD^2^ score for assessing the risk of 90-day stroke after TSI (Z = 2.115, P = 0.0344). There is evidence confirming that taking into account DWI and TIA etiology improves the prediction of the early risk of stroke after TIA [7,21,22,28,29]. The RRE-90 scale pays much more attention than the ABCD^2^ score to imaging and etiology. Therefore, the RRE-90 score might be more suitable for TSI patients, and could help clinicians to select high-risk patients for hospitalization, economize on medical resources, and determine individualized secondary prevention strategies to reduce the risk of stroke after TSI. However, although MRI and, in particular, DWI is markedly superior to other imaging techniques in the evaluation of ischemic lesions and in differentiating acute infarcts from chronic lesions [30], it suffers from limited accessibility and applicability because of its high cost. Moreover, the ABCD^2^ score is useful as an initial screening tool for use by non-neurologists to triage TIA patients rapidly. In contrast to ABCD^2^, the RRE needs more time and is suitable for neurologists, thus limiting its utility.

In comparison with the previous retrospective research [14], our study is a prospective cohort study. The previous study evaluated the predictive value of the RRE-90 scale at 7 days; our study extends the utility to 90 days. Hence, if the sample size is further enlarged and validated for different time windows, RRE could be applied to any patient with ischemic lesions in the brain, whether with persistent or transient symptoms. In this respect, our study provides information supplementary to the new tissue-based definition of ischemic stroke.

Several potential limitations of this study need to be addressed. First, the small sample size affected the sensitivity and specificity of the prognostic score. Second, because this was a single-center study, selection bias was unavoidable. Moreover, given the character of our hospital (a teaching general hospital), some TSI patients with mild symptoms and those in poor economic circumstances might not be hospitalized. Hence, the predictive value of RRE may have varied according to the study design, data collection method, and clinical setting. Additional multicenter validation of this score is therefore required. Third, although there is evidence confirming that RRE-90 bears high potential to predict not only early recurrence but also death and progression after ischemic stroke [31], subsequent research has not proceeded because of the small sample size.

## Conclusion

Our research shows that the 90-day predictive value of the RRE score is higher than that of the ABCD^2^ score in TSI patients. In terms of clinical practice, the RRE-90 scale has the potential to improve the care and outcomes of TSI patients.

## Supporting Information

S1 Data Raw data of the present study. (XLSX)
